# N–H⋯O versus O–H⋯O: density functional calculation and first principle molecular dynamics study on a quinoline-2-carboxamide N-oxide

**DOI:** 10.1007/s00894-015-2587-3

**Published:** 2015-02-19

**Authors:** Aneta Jezierska

**Affiliations:** Faculty of Chemistry, University of Wrocław, ul. F. Joliot-Curie 14, 50-383 Wrocław, Poland

**Keywords:** N-oxide, Intramolecular hydrogen bond, Density functional theory, Atoms in molecules, Car-Parrinello molecular dynamics, Gas and crystalline phase

## Abstract

N-oxide-type compounds are the object of current research interest due to the presence of resonance-assisted N–H⋯O hydrogen bonds. Here, the metric and spectroscopic parameters of N-methyl-quinoline-2-carboxamide 1-oxide were computed on the basis of density functional theory and Car-Parrinello molecular dynamics. Computations were performed in vacuo and in solid state; for both phases additional simulations with Grimme’s dispersion correction were carried out. The approaches used were able to reproduce correctly the structural aspects of the studied compound and shed more light on the hydrogen bonding with special focus on bridge proton mobility. Proton transfer phenomena were found not to occur in the investigated compound, and the bridge proton was localized to the donor site. This observation is in agreement with the classical theory of the acidity of donor–acceptor sites. The presence of hydrogen bonding was confirmed using atoms-in-molecules theory. The computational results were compared with available experimental data.

**Electronic supplementary material:**

The online version of this article (doi:10.1007/s00894-015-2587-3) contains supplementary material, which is available to authorized users.

## Introduction

**Fig. 1:**
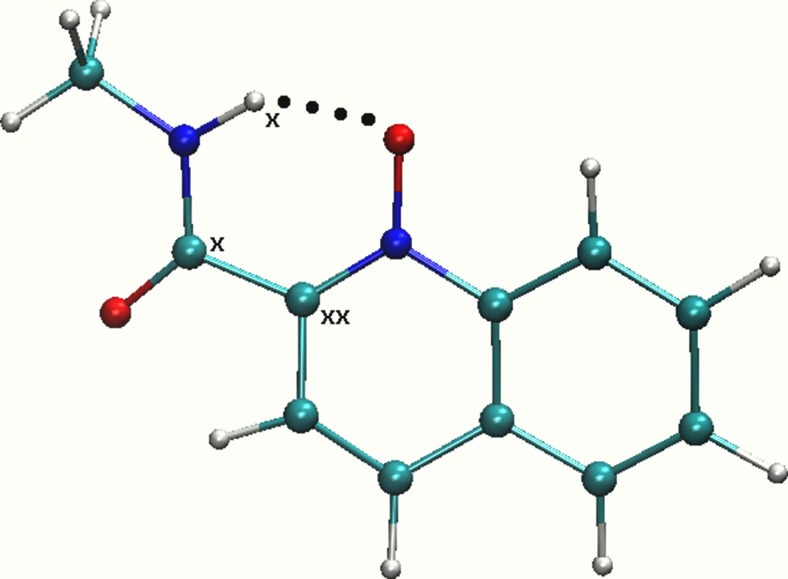
Computational model of N-methyl-quinoline-2-carboxamide 1-oxide used for gas phase static density functional theory (DFT) simulations and Car-Parrinello molecular dynamics (CPMD) in vacuo. *Dotted line* Intramolecular hydrogen bond (H-bond). Atoms: *White* hydrogen, *blue* nitrogen, *red* oxygen, *cyan* carbon. *X* Atoms significant for NMR study

Intra- and inter-molecular hydrogen bonds (H-bonds) have been the subject of many studies during recent decades. As this field has developed, beside well-defined conventional H-bonds, one can find so-called unconventional H-bonds (see e.g., [1–6]), which play important roles in many processes at a molecular level (see e.g., [7–9]). The focus of the current study is one of the group of conventional intramolecular H-bonds. Among this group one can find charge-assisted, resonance-assisted [10] or ionic [11] H-bonds. Here, continuing our previous research related to N-oxide-type compounds, N-methyl-quinoline-2-carboxamide 1-oxide was investigated. This compound possesses a resonance-assisted N–H⋯O H-bond (see Fig. 1) confirmed experimentally in 2009 by Kamiński et al. [12]. Previous studies of quinoline-2-carboxamides and their N-oxides from the 1970s to 1980s excluded the existence of the intramolecular N–H⋯N H-bond in the primary quinoline-2-carboxamides [13, 14], while a recent study [12] described the N–H⋯N contacts as very weak, non-linear H-bonds, weak enough to be not decisive in the formation of structural motifs in the crystal (intermolecular H-bonds were found to be more important [12]). However, the N-oxide function strengthens the intramolecular H-bond significantly, making it the primary structural factor in the solid state. For example, molecules belonging to the secondary carboxamides were connected only by weak intermolecular contacts [12]. Therefore, following recent findings related to H-bonding presence and character, the current report presents computations based on static and molecular dynamics models to shed more light on the presence and dynamics of H-bonds.

N-oxides are important in many areas of contemporary research. They have been identified as potential anticancer [15] and antimalarial [16] drugs or reductors of bacterial deposition [17], whereas heterocyclic N-oxides have been classified as a class of antitumor agents that selectively kill the hypoxic cells found in solid tumors [18]. N-oxides also play a significant role in materials chemistry. For example, pretreatment of lignocellulosic biomass by N-methylmorpholine-N-oxide (NMMO)—a solvent used in the textile industry to dissolve cellulose for production of regenerated cellulose fibers—was noticed to enhance significantly enzymatic saccharification and fermentation [19]. In the new “click chemistry” nitrile N-oxides found applicability in polymer synthesis [20]. The interfacial and structural features of dimethyldodecylamine-N-oxide (DDAO) micelles were studied due to their usefulness and practical applications in the food, cosmetics and pharmaceutical industries [21]. Pyridine and quinoline N-oxides were employed in regioselective and enantioselective catalysis [22, 23]. Finally, and most relevantly for this study, a class of carboxamide-pyridine N-oxides was proposed as new heterosynthons for crystal engineering with possible use in the design of pharmaceutical cocrystals [24]. This heterosynthon is based on the increased acceptor strength of anionic (N-oxide) oxygen, and prefers formation of heterodimers over conventional homodimers.

As shown above, N-oxides are well-recognized compounds with very diverse applicability and valuable chemistry. Moreover, N-oxide derivatives are a class of compounds in which various types of intramolecular H-bond can be formed. Some recent examples include picolinic acid N-oxide [25] or Mannich base N-oxides [26, 27], which possess strong O–H⋯O H-bonds. Their character manifests itself in unique spectroscopic signatures characteristic of very strong H-bonds: broad absorptions, large red shifts and strange isotope effects. This study aimed to determine computationally the properties of another type of H-bond: N–H⋯O, with emphasis on similarities and differences with the O–H⋯O N-oxides. Quinoline N-oxides contain two fused rings with aromatic features; the presence of a nitrogen atom in one of the rings and the oxygen atom connected to it give them a very interesting chemical composition, which provides the possibility of introducing modifications by introduction of various substituents, e.g., by introduction of intramolecular H-bonds, which results in quasi-ring formation. Indeed, this was the basis of the abovementioned proposal to use pyridine N-oxides as a heterosynthon in co-crystallization [24]. Our previous research related to N-oxide-type compounds [26] revealed the presence of very short and strong type of intramolecular H-bonding defined as a low-barrier-hydrogen-bond (LBHB). Car-Parrinello molecular dynamics (CPMD) was performed for the Mannich base N-oxide and it was found that the bridge proton is delocalized strongly and shared almost equally between the donor and acceptor atoms. We demonstrated that, in the case of the O–H⋯O H-bond in this particular N-oxide, the potential energy surface (PES) is flat and the inclusion of quantum effects for nuclear motion plays an important role. The computations performed to determine one- and two-dimensional potential of mean force (Pmf) support the proposed model of a single, flat, and almost symmetrical potential well of proton motion, which shows a high degree of proton delocalization. Another type of intramolecular H-bond investigated previously concerns aromatic Schiff and Mannich bases [28] where O–H⋯N type bonding is present. It was found that, in the studied Schiff base, the bridge proton is more labile and proton transfer phenomena can occur. In the case of the Mannich base, the bridge proton is localized on the donor site. Other examples of intramolecular hydrogen bonds have been investigated, e.g., 2-hydroxy-N-methylbenzamide (O–H⋯O) and 2-hydroxy-N-methylthiobenzamide (O–H⋯S) [29]. Here, special attention was paid to differences relating to the various acceptor atoms (O or S), which influence bridge proton dynamics. In the case of 2-hydroxy-N-methylbenzamide, the bridge proton is delocalized strongly in the crystalline phase, and therefore spontaneous proton transfer was observed. The opposite situation was noted for 2-hydroxy-N-methylthiobenzamide, i.e., the bridge proton is localized on the donor site in both phases. Another example of an infrequently studied O–H⋯S H-bond was investigated in 3-mercapto-1,3-diphenylprop-2-en-1-one [30]. The position of the bridge proton on the donor site was found to be more dominant in the solid state than in the gas phase. Our findings indicate a lack of proton transfer under the conditions applied and on the first principle molecular dynamics (MD) time-scale. As has been shown, various types of H-bonding have been taken into account in our research, expanding our knowledge of bridge proton dynamics and forming the backdrop for further research in this area.

In the work reported in this paper, static models and first-principle MD were employed for simulations of N-methyl-quinoline-2-carboxamide 1-oxide in vacuo and in the crystalline phase. Throughout the study, the term “static DFT” is used to differentiate standard techniques of geometry optimization (resulting in a “static” picture of the minimum on the PES) from the dynamic picture obtained from the MD scheme. The results obtained include dispersion effects computed with density functional theory (DFT) [31] and CPMD [32]. The computations in two phases allowed a more detailed study of the mobility of the bridge proton and its influence on the molecular features of the investigated N-oxide. Ab initio MD was employed to analyze not only metric parameters as a function of time, but also to draw a picture of spectroscopic features to make some comparisons with available experimental data [12]. Therefore the main aims of this work cover aspects related to the metric and spectroscopic parameters of time-evolution in picosecond time-scale obtained from CPMD simulations. Because N-oxides are used widely in many areas of human life, it is worthwhile pursuing the rational design of new compounds based on well-recognized parent compounds. The paper is organized as follows: a section on Computational methods is followed by the Results and discussion, with some final remarks given in the Conclusion.

## Computational methods

### Static density functional theory

Static DFT [31] was applied to develop static gas phase models for N-methyl-quinoline-2-carboxamide 1-oxide. On the basis of the results obtained, geometric, spectroscopic and topological properties were analyzed. Figure 1 presents the model structure used for simulations. Three types of functionals were applied, namely B3LYP [33, 34], PBE [35] and wB97xD [36]. In order to describe weak interactions, Grimme’s dispersion correction was incorporated [37]. A basis set of the Pople type [6-311++G(d,p)] [38] was chosen for structure optimization. Geometry minimization was performed according to standard procedures implemented in the Gaussian 09 suite of programs [39]. Harmonic frequencies were calculated to confirm that the structures obtained correspond to minima on the PES. Nuclear magnetic resonance (NMR) properties were calculated using the gauge-independent atomic orbital (GIAO) method [40]. The output of NMR computations includes chemical shifts for all the nuclei in the molecule, but here we focus on the signatures related to the hydrogen atom involved in intramolecular H-bond formation and the two carbon atoms involved in quasi-ring formation. The chemical shifts were calculated with all functionals used and with 6-311++G(d,p), 6-311++G(2d,2p) and 6-311++G(3df,3pd) basis sets. For the two largest basis sets, single point NMR simulations were performed using the structure optimized with the 6-311++G(d,p) basis set. Additionally, geometry optimization and NMR chemical shifts were obtained for tetramethylsilane (TMS), which served as a reference standard. The chemical shifts were calculated according to the equation:

$$ {\mathrm{d}}_{\mathrm{H}}={{\mathrm{d}}_{\mathrm{H}}}^{\mathrm{TMS}}-{\mathrm{d}}_{\mathrm{H}} $$

where δ_H_^TMS^ and δ_H_ are isotropic shielding constants in the reference and investigated compound, respectively. Next, atoms in molecules (AIM) theory [41] was applied to study the topology of the intramolecular H-bond. The following topological parameters were calculated for this purpose: electron density, ρ and its Laplacian, ∇^2^ρ, computed at bond critical points (BCPs). The Gaussian 09 suite of programs [39] was used to obtain wavefunctions for the AIM analysis. The three functionals mentioned above and the 6-311++G(d,p) basis set were used. The data obtained provided additional information on the nature of the chemical bonding present in the investigated N-oxide type compound. The properties of the intramolecular H-bond were examined by application of Popelier’s criteria [42]. For covalent bonds, the electron density at the BCP is of the order of 0.1 a.u. For non-covalent interactions, e.g., weak H-bonds and van der Waals interactions, it is lower by about 0.01 a.u. or even less. The AIM analysis was carried out using the AIMPAC package [43].

### Car-Parrinello molecular dynamics

DFT-based Car-Parrinello molecular dynamics (CPMD) [32] were applied to study the geometric and spectroscopic properties of N-methyl-quinoline-2-carboxamide 1-oxide. The simulations were performed in the crystalline phase and in the gas phase to evaluate environmental influences on the investigated molecule. Dispersion corrections according to Grimme’s scheme [37] were also incorporated to reproduce weak interactions contributions to geometric and spectroscopic features. The first step comprised structure minimization performed using the Schlegel approximation for the initial Hessian [44]. The Perdew-Burke-Ernzerhof (PBE) functional [35] and Troullier-Martins norm-conserved pseudopotentials [45] were applied throughout the CPMD computations. For the plane-wave basis set, a kinetic energy cutoff convergence with various values were tested (range: from 70 Ry to 120 Ry) in both phases. In the next step, CPMD simulations were performed. The energy cutoff value chosen for the MD simulation was 100 Ry and the time-step was set to 3 a.u. for both phases. The applied fictitious electron mass parameter (EMASS) was equal to 400 a.u. The simulations were carried out at room temperature (297 K) and controlled using the Nosé thermostat [46, 47].

In the solid state model, an experimental monoclinic unit cell with *a* = 10.2715 Å, *b* = 7.7881 Å, *c* = 11.6200 Å, and *β* = 93.772° with *Z* = 4 [12] was used during the computations. The periodic boundary conditions (PBCs) were applied with electrostatic summations for the eight nearest neighbors in each direction (TESR = 8). The CPMD simulations were performed with Γ point approximation. The data collection time was ca. 28 ps for simulations with and without Grimme dispersion correction. The initial simulation time (2,000 steps) was taken as the equilibration phase and was not taken into consideration during the post-processing analyses in both phases. The gas phase CPMD calculations were performed in a cubic cell of *a* = 20 Å with the Hockney periodic image removal scheme applied. The size of the unit cell was dictated by the need to avoid artifacts at the cell boundary. The data collection time was ca. 28 ps for classical CPMD and ca. 16 ps for simulations with Grimme’s dispersion correction. The CPMD trajectories were collected using the CPMD v3.15.3 suite of programs [48]. Post-processing analyses were performed to study the time-evolution of the interatomic distances involved in the intramolecular hydrogen bond. The N⋯O, N–H and H⋯N distances and NHO valence angle were analyzed. The analyses were performed on the basis of drawing a time-evolution scheme of the distances as a function of simulation time as well as using 2D histograms. Subsequently, Fourier transformation was used to convert the atomic velocity and dipole moment values into vibrational features in the frequency domain. In order to obtain the power spectra of atomic velocity, a home-made script was used whereas for the spectra based on dipole moments the script written by Harald Forbert (http://www.cpmd.org/download) was applied. Graphics were prepared within the framework of the VMD [49], Mercury [50] and Gnuplot [51] programs.

## Results and discussion

The specificity of H-bonding continues to be investigated, both experimentally and computationally, with many open questions concerning the role of H-bonding and its constitution remaining unanswered. Here, a detailed analysis of an intramolecular hydrogen bond in N-methyl-quinoline-2-carboxamide 1-oxide is presented with special attention paid to the dynamics of the hydrogen bridge. Available experimental data was used as a reference for the computations performed [12]. In addition, in analyzing the results obtained for the N–H⋯O H-bond in the studied N-oxide, some reference will be made to the O–H⋯O hydrogen bridge studied previously [26] as well as to experimental infrared (IR) data on analogous pyridine N-oxides [24].

The model used for computations is presented in Fig. 1. Selected metric parameters obtained on the basis of various DFT-based approaches are listed in Table S1 of the Online Resource. As shown, the geometric parameters of the intramolecular H-bond are reproduced correctly. Comparing with X-ray measurements, the N⋯O interatomic distance is elongated by ca. 0.028 Å–0.03 Å according to computations performed on the basis of classical DFT theory. The CPMD average distances for the solid state simulations are in line with teh static DFT results, whereas the gas phase CPMD provided an elongated N⋯O average distance by ca. 0.05 Å. The N–H bond length was reproduced in a similar manner by all applied methods. The calculated bond length was ca. 1 Å and seemed to be reproduced correctly. The N–H bond length can be compared with the N–H bond lengths present in ammonia, which has been reported as 1.012 Å [52]. Experimental measurements (X-ray) for the N–H bond length present in the investigated N-oxide gave a bond length of 0.907 Å, which is in good agreement with computations, taking into account the fact that the proton position in the intramolecular H-bridge was found with well-known approximations associated with X-ray measurement specificity. All applied methods reproduced the H⋯O bond length relatively well. The bond length was shortened in all applied methods. The largest discrepancy was found for PBE/6-311++G(d,p), PBE-D3/6-311++G(d,p) and CPMD (solid state) average. The difference between experimental and calculated values was found to be 0.073 Å, 0.062 Å and 0.067 Å. The valence bond between atoms involved in hydrogen bond formation was also computed. The values obtained correspond well with experimental data. The best agreement was found for PBE/6-311++G(d,p), as presented in Table S1 of the Online Resource.

Another issue tested during the simulations was the influence of cutoff value on reproduction of the metric parameters involved in the intramolecular H-bond (see Table S2 of the Online Resource). The simulations were performed in vacuo and in the crystalline phase. In addition, to include dispersion effects, Grimme’s correction was performed. As shown by the results, increasing the plane-wave number does not change the values obtained significantly. Concerning the crystalline phase simulations, the best agreement with the experimental result for the N⋯O interatomic distance was found for the 100 Ry cutoff; this value was further used for the CPMD simulations. The N–H bond length was elongated slightly compared with the experimental data. Therefore, following previous observations, one could expect the H⋯O bond length to be shortened compared with experimental findings. The value of the valence angle was reproduced pretty well and similarly with all tested cutoff values.

The NMR computations were performed with the assistance of various functionals, including dispersion effects and basis sets. Here, only three atoms from the studied N-oxide were analyzed (see Fig. 1 for details). Two carbon atoms involved in quasi-ring formation and the bridge hydrogen atom were chosen as the most important and representative for this part of the study. The results obtained are presented in Table 1. The computations were performed using the popular GIAO method. A relatively good agreement with experimental data [12] was found for all applied methods. However, the best performance was noted for the PBE and PBE-D3 functionals in the case of carbon atoms. Closest to the experimental data for the C_xx_ atom (see Fig. 1 for notation) was the result obtained at PBE-D3/6-311++G(2d,2p) level of theory. In the case of the C_x_ atom, again the PBE-D3 functional proved to be the best, but the basis set was 6-311++G(3df,3pd). For the hydrogen atom involved in intramolecular H-bond formation, all methods applied reproduced the chemical shift in a similar manner with small discrepancies compared to the experimental findings. A significant deshielding of the bridge proton was connected to the strength of the intramolecular H-bond; however, in the O–H⋯O analog [26], the experimental δ_H_ was 17.8 ppm, as compared to 11.43 ppm in the currently studied compound. For a series of smaller (one-ring) pyridine N-oxides with N–H⋯O bridges, the δ_H_ values lies in the range 11.45–11.81 ppm; however, introduction of two N–H donors forming competitive bridges to the same N-oxide oxygen acceptor, slightly decreases the bridge strength; δ_H_ was found to be 10.81–11.15 ppm [22]. We conclude that the hydrogen bridge in our case is of similar strength as that of the analogous carbamate pyridine N-oxides [22, 24], and much weaker than in the case of O–H⋯O [26].

**Table 1:** Comparison of experimental [12] and calculated nuclear magnetic resonance (NMR) chemical shifts of the hydrogen atom involved in intramolecular hydrogen bond (H-bond) formation and the two carbon atoms involved in quasi-ring formation of N-methyl-quinoline-2-carboxamide 1-oxide

	C_xx_ (ppm)	C_x_ (ppm)	H_x_ (ppm)
Experimental [12]	137.5	162.1	11.43
Level of theory			
B3LYP/6-311++G(d,p)	145.87	166.16	10.93
B3LYP/6-311++G(2d,2p)	145.44	166.22	11.29
B3LYP/6-311++G(3df,3pd)	146.27	167.24	11.61
B3LYP-D3/6-311++G(d,p)	146.00	166.25	10.87
B3LYP-D3/6-311++G(2d,2p)	145.58	166.31	11.24
B3LYP-D3/6-311++G(3df,3pd)	146.40	167.31	11.55
PBE/6-311++G(d,p)	140.37	160.17	11.27
PBE/6-311++G(2d,2p)	140.09	160.24	11.65
PBE/6-311++G(3df,3pd)	141.11	161.64	11.96
PBE-D3/6-311++G(d,p)	140.07	161.10	11.24
PBE-D3/6-311++G(2d,2p)	139.81	161.20	11.66
PBE-D3/6-311++G(3df,3pd)	140.82	162.46	11.97
wB97xD/6-311++G(d,p)	144.93	165.47	10.98
wB97xD/6-311++G(2d,2p)	144.42	165.57	11.34
wB97xD/6-311++G(3df,3pd)	145.4	166.67	11.64

Following investigation of the intramolecular H-bond, a topological analysis of electron density in the framework of AIM theory was performed. The values of electron density and its Laplacian at the N–H and H⋯O BCPs are presented in Table 2. The results were obtained on the basis of five functionals (with and without Grimme’s correction). A previous AIM study was performed at the DFT B3LYP/6-311++G(d,p) level of theory [53] and the data found was in agreement with the current results. The inclusion of dispersion effects during the simulations and later during the electron density partitioning did not introduce any significant changes to the results obtained. Moreover, the results obtained at BCPs for the hydrogen bonding path follow the ranges estimated by Koch and Popelier [42]. The properties of the H⋯O BCP, especially the rather high electron density, indicate that this bridge is medium–strong. Thus, the properties of the N–H⋯O intramolecular H-bond were reproduced in agreement with the experimental findings [12] and previous computations [53].

**Table 2:** Electron density and its Laplacian evaluated at bond critical points (BCPs) of the intramolecular hydrogen bridge studied at various levels of theory—density functional theory (DFT) static models. *AIM* Atoms in molecules

Level of theory	AIM properties	N–H	H⋯O
B3LYP/6-311++G(d,p)	ρ(e*a_0_^−3^)	0.3304	0.0392
	∇^2^(ρ)(e*a_0_^−5^)	−1.7424	0.1389
B3LYP-D3/6-311++G(d,p)	ρ(e*a_0_^−3^)	0.3307	0.0387
	∇^2^(ρ)(e*a_0_^−5^)	−1.7452	0.1380
PBE/6-311++G(d,p)	ρ(e*a_0_^−3^)	0.3185	0.0430
	∇^2^(ρ)(e*a_0_^−5^)	−1.5835	0.1385
PBE-D3/6-311++G(d,p)	ρ(e*a_0_^−3^)	0.3186	0.0420
	∇^2^(ρ)(e*a_0_^−5^)	−1.5867	0.1368
wB97xD/6-311++G(d,p)	ρ(e*a_0_^−3^)	0.3309	0.0396
	∇^2^(ρ)(e*a_0_^−5^)	−1.8036	0.1423

**Fig. 2:**
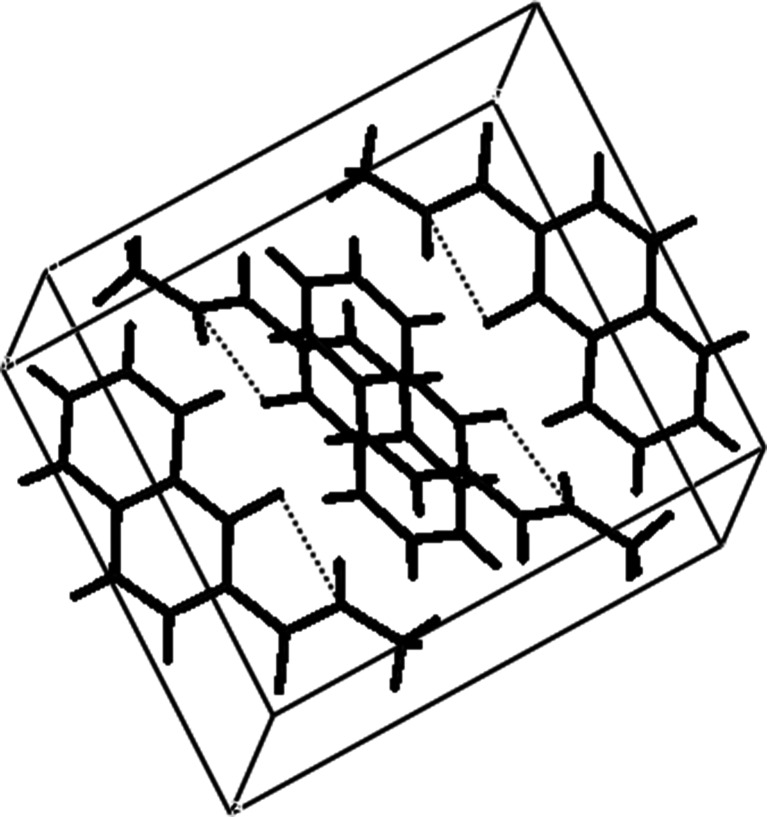
Model of N-methyl-quinoline-2-carboxamide 1-oxide used for Car-Parrinello molecular dynamics (CPMD) simulations in the crystalline phase. *Dotted lines* Intramolecular H-bonds

The model used for crystalline phase simulations on the basis of CPMD is presented in Fig. 2. As mentioned in the Computational methods section, the CPMD simulations were performed in vacuo and in the crystalline phase. Figure S1 of the Online Resource presents the results of time-evolution of interatomic distances of atoms forming the intramolecular H-bond. At the tested time-scale and under the conditions applied, proton transfer phenomena did not occur. The bridge hydrogen is well-defined at the donor site.

**Fig. 3:**
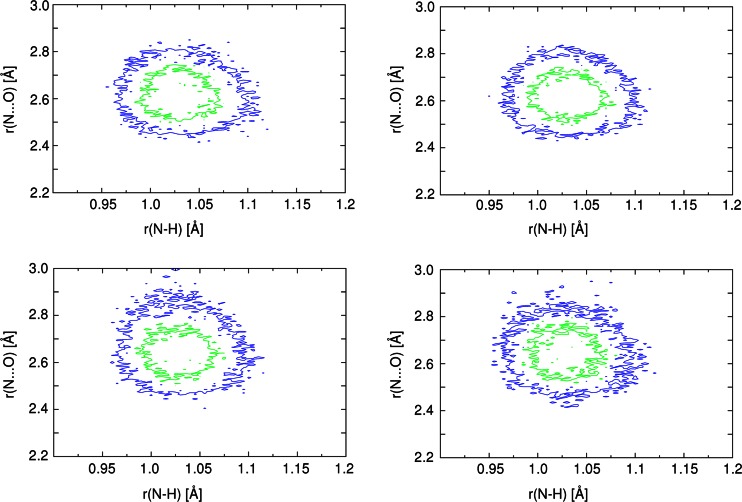
Joint probability density for the N–H and N⋯O distances from CPMD simulations of N-methyl-quinoline-2-carboxamide 1-oxide within first-principles molecular dynamics (FPMD, *left column*) and with inclusion of dispersion effects (*right column*). Upper graphs Results obtained for the crystalline phase, lower graphs results of simulations in vacuo. Probability density isocontours: *inner* 15 Å^−2^, *outer* 5 Å^−2^

One of the most important and useful advantages of the CPMD method is the capability to estimate both the dynamics of H-bonding and the mobility of the bridge hydrogen. Figure 3 shows histograms of the N⋯O and N–H interatomic distances also obtained as the CPMD results in both phases. As shown, inclusion of the dispersion correction does not change the overall picture of the hydrogen bridge dynamics. The interatomic N⋯O distance during the CPMD run in the solid state was found to be between 2.45 Å and 2.85 Å. The changes in N–H bond length during the MD simulations cover the range between 0.96 Å up to ca. 1.12 Å. In the gas phase the N⋯O interatomic distance was found to be 2.45–2.9 Å. The N–H bond length does not change dramatically (0.95 Å–1.11 Å), but a slight elongation was noted when dispersion effects were included (lower right panel in Fig. 3). Summarizing the hydrogen bridge dynamics and the bridge hydrogen mobility, it has been shown that similar results were obtained for both phases. In the crystalline phase, the H-bond dynamics could be limited by the presence of neighboring molecules and crystal field effects, whereas in the gas phase all degrees of freedom were preserved. In the case of the studied N-oxide the H-bond is stabilized by the chemical composition of the compound, because no significant differences in the bridge dynamics were noted and there was no clear and visible influence on other possible variables.

The metric parameters of the intramolecular bridge are relatively close to the values found in a typical strong, resonance-assisted H-bond in two chloro-derivatives of *o*-hydroxyacetophenone (2.559 and 2.567 Å) [54], even if the current compound presents a weaker N–H⋯O bond type, while the acetophenone derivatives possess stronger O–H⋯O contacts. However, how does the studied case compare with other N-oxide derivatives? The relevant comparison with available experimental data for picolinic (PANO) and quinaldic (QANO) acid N-oxides [55–57] is presented in Table 3. QANO has a slightly longer O⋯O distance than PANO, but both are very short in comparison with the current compound, which has a mid-strength H-bond. This is also reflected in the much larger deviation from linearity in the bridge of the studied compound. Despite the same formal shape of the quasi-ring formed by the hydrogen bridge, the difference between the properties of the –OH and –NH moieties as proton donors is large enough to change the type of bridge from a very short and strong bridge (PANO, QANO) to one of mid-strength (the current case). This fact is also reflected in the IR signature of the current compound, as discussed below. It should be stated that CPMD is able to reproduce the effect of the environmental electrostatic crystal field on the N–H⋯O bridge: the solid-state N⋯O distance is 0.03 Å shorter than in the gas phase (and only 0.02 Å longer than the experimental result). Table 3 also highlights another important issue: the inclusion of dispersion corrections in solid-state computations does not improve the results for the intramolecular H-bond of the studied compound. The use of such corrections is, however, generally recognized as necessary for DFT-based simulations of structures of molecular crystals, which are stabilized, sometimes to a large degree, by dispersion interactions.

**Table 3:** Comparison of interatomic distances of atoms involved in the intramolecular H-bond for N-methyl-quinoline-2-carboxamide 1-oxide (NQCO) and picolinic (PANO) or quinaldic (QANO) acid N-oxides. Calculations for NQCO are averaged values from CPMD runs (this work) and experimental values taken from [12, 55, 57]

Compound	Bridge type	D–H [Å]	H⋯A [Å]	D⋯A [Å]	D–H⋯A [°]
PANO	O–H⋯O				
X-ray [55]		1.04(3)	1.42(3)	2.425(2)	159(3)
X-ray [57]		–	1.445	2.422	–
QANO	O–H⋯O				
X-ray [55]		0.98(3)	1.48(3)	2.435(2)	161(2)
NQCO	N–H⋯O				
X-ray [12]		0.91(2)	1.84(2)	2.598(1)	139(2)
CPMD, gas		1.0320	1.8232	2.6521	135.34
CPMD + dispersion, gas		1.0319	1.8196	2.6498	135.40
CPMD, solid		1.0339	1.7752	2.6219	136.84
CPMD + dispersion, solid		1.0339	1.7835	2.6251	136.30

**Fig. 4:**
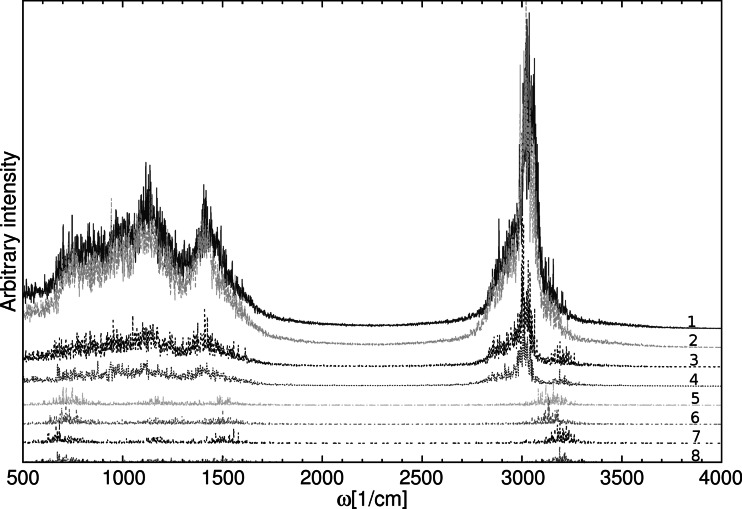
Comparison of atomic velocity power spectra of all atoms and the bridge proton of N-methyl-quinoline-2-carboxamide 1-oxide as a result of CPMD simulations within classical nuclear dynamics (FPMD) and with inclusion of dispersion effects in the crystalline phase and in vacuo. Intensities are in arbitrary units, whereas wavenumbers correspond to the actual vibrational properties of the studied compound. Numbers visible in the spectra indicate: *1* all atoms in the crystalline phase, *2* all atoms in the crystalline phase with dispersion correction, *3* all atoms in the gas phase, *4* all atoms in the gas phase with dispersion correction, *5* bridge hydrogen in the crystalline phase, *6* bridge hydrogen in the crystalline phase with dispersion correction, *7* bridge proton in the gas phase, *8* bridge proton in the gas phase with dispersion correction

The IR spectroscopic features were investigated on the basis of atomic velocity and dipole moments power spectra. The computed power spectra of atomic velocity obtained are shown in Fig. 4. The spectra were obtained for results where the dispersion corrections of Grimme were included as well as trajectories without Grimme correction. Both phases were taken into account for the analysis. Additional IR computed data are included in the Online Resource (Figs. S2, S3, S4) where, in addition to atomic velocity power spectra, dipole dynamics results are also presented. As seen in Fig. 4, the inclusion of dispersion effects did not change the IR features. Therefore, for this kind of compound, with quite a rigid chemical constitution, it can be concluded that dispersion effects do not play a significant role. The Fourier transform of atomic velocity enables a division of bands in the computed spectra thus the bridge proton was selected and its bands in the spectra are shown as separated graphs. In both phases, two characteristic regions with broad adsorption could be distinguished: for solid state, 500 cm^−1^–170 cm^−1^ and 3000 cm^−1^–3300 cm^−1^, whereas for the gas phase 500 cm^−1^–1700 cm^−1^ and 2900 cm^−1^–3400 cm^−1^. The most intense motions were found at ca. 3150 cm^−1^ in the solid state and at ca. 3200 cm^−1^ for the gas phase. In addition, there were some shifts in the solid state toward lower wavenumbers compared to those obtained in the gas phase—an effect showing the moderate impact on the bridge properties of the crystal electrostatic field. Vibrational data for analogous N-oxides are scarce; however, picolinamide N-oxide (containing intramolecular N–H⋯O bonds) exhibits N–H bands at 3103 and 3250 cm^−1^, whereas the respective band positions for nicotinamide N-oxide are 3298 and 3144 cm^−1^, and for isonicotinamide N-oxide, 3350 and 3153 cm^−1^ [24]. The two latter compounds are not able to form intramolecular bridges. Our computational results are in line with experimental data for smaller pyridine N-oxides and indicate that the impact of N–H⋯O H-bonding on vibrational features of the N–H group is much smaller than in the case of the OH group in similar O–H⋯O-containing N-oxides [26, 56].

In summarizing H-bond features in N-oxide type compounds, it is very difficult to make any general conclusions. The investigated compound has a well-preserved chemical constitution at the ground state at the time-scale investigated. The present hydrogen bridge stabilizes the quasi-ring, therefore no other conformers and events, e.g., bond breaking, were observed during the CPMD runs. Therefore, the experimental findings concerning the molecular structure (the presence and strength of H-bonding) once more supported the computational findings.

## Conclusions

In the current study, N-methyl-quinoline-2-carboxamide 1-oxide was investigated. For this purpose, various theoretical approaches were applied. Computational findings were compared with experimental data. The geometric parameters involved in the intramolecular N–H⋯O hydrogen bond were reproduced correctly at all applied levels of theory. The NMR spectroscopic signatures for the hydrogen atom involved in H-bond formation as well as two carbons atoms from the quasi-ring were found to be in good agreement compared to experimental chemical shifts. The presence of H-bonding was confirmed by the topological analysis of electron density on the basis of the AIM method. Bridge proton mobility and hydrogen bridge dynamics were investigated using CPMD methods. According to expectations, the proton transfer phenomenon did not occur, showing a different character of the H-bond compared to the O–H⋯O hydrogen bond in N-oxides studied previously [26]. The bridge proton does not exhibit high mobility and it is localized at the donor site. The IR spectra obtained as a result of Fourier transform of atomic velocity and dipole moment reveal the spectral bands related to the bridge proton as well as to all atoms in the molecule. As shown, the wavenumbers related to proton motion are in agreement with experimental findings for H-bonds, confirming spectrally the presence of bonding in the molecule. Dispersion effects do not play a significant role in bridge proton delocalization in this compound. No dramatic changes were observed when analyzing the metric and spectroscopic signatures and comparing them with computations performed without Grimme’s correction. Summarizing, because of its chemically rather rigid constitution, stabilized further by the quasi-ring formation, the studied N-oxide type compound could be further modified by the introduction of inductive and steric effects in the carboxamide moiety as well as by substitution of the fused ring in future structure–property related studies. Such studies could be motivated by the presence of excited-state-induced proton transfer phenomena, registered for extended aromatic systems (e.g., quinoline derivatives) and potentially useful in such applications as molecular optical switches.

## Electronic supplementary material

Below is the link to the electronic supplementary material.

ESM 1 (PDF 2915 kb)
