# Supplementary material for: N–H⋯O versus O–H⋯O: density functional calculation and first principle molecular dynamics study on a quinoline-2-carboxamide N-oxide
Source: J Mol Model. 2015 Feb 19;21(3):47. doi: 10.1007/s00894-015-2587-3 (PMC4333232; doi:10.1007/s00894-015-2587-3)
Supplement: Supplementary file 1 — (PDF 2915 kb) [file 894_2015_2587_MOESM1_ESM.pdf]

# Supplementary materials

# N-H...O versus O-H...O: Density functional calculation and first principle molecular dynamics study on a quinoline-2-carboxamide N-oxide

Aneta Jezierska*

University of Wrocław, Faculty of Chemistry, ul. F. Joliot-Curie 14, 50-383 Wrocław, Poland

*** Correspondence should be addressed to:** Aneta Jezierska, e-mail: aneta.jezierska@chem.uni.wroc.pl, Tel.: +48 71 3757 224, Fax: +48 71 3282 348

**Table of contents**

I. **Figure 1SI.** Time-evolution of the interatomic distances of the atoms involved in the hydrogen bridge formation. Results obtained from CPMD simulation within classical nuclear dynamics (left) and within inclusion of dispersion effects (right) in crystalline phase (upper graphs) and *in vacuo* (lower graphs).

II. **Figure 2SI.** Power spectra of the atomic velocity of all atoms and the bridge proton of N-methyl-quinoline-2-carboxamide 1-oxide as a result of the CPMD simulations in the crystalline phase (upper spectra) and *in vacuo* (lower spectra). The intensities are given in arbitrary units, whereas the wavenumbers correspond to the actual vibrational properties of the studied compound.

III. **Figure 3SI.** Power spectra of the atomic velocity of all atoms and the bridge proton of N-methyl-quinoline-2-carboxamide 1-oxide as a result of the CPMD simulations with Grimme's dispersion correction in the crystalline phase (upper spectra) and *in vacuo* (lower spectra). The intensities are given in arbitrary units, whereas the wavenumbers correspond to the actual vibrational properties of the studied compound.

IV. **Figure 4SI.** Predicted IR spectrum of N-methyl-quinoline-2-carboxamide 1-oxide. Results obtained from the CPMD simulation in the crystalline phase and *in vacuo*. Intensities, in arbitrary units, are proportional to the observable IR intensities.

V. **Table 1SI.** Comparison of the experimental X-ray (Ref. 12) and computed interatomic distances of atoms involved in the intramolecular hydrogen bond formation obtained

for N-methyl-quinoline-2-carboxamide 1-oxide. Static DFT and CPMD in the crystalline phase and *in vacuo*.

VI. **Table 2SI.** Comparison of the experimental (Ref. 12) and computed geometric parameters (crystalline phase and *in vacuo*) of the studied N-H…O hydrogen bond: investigations of the influence of the cutoff value on the geometric parameters.

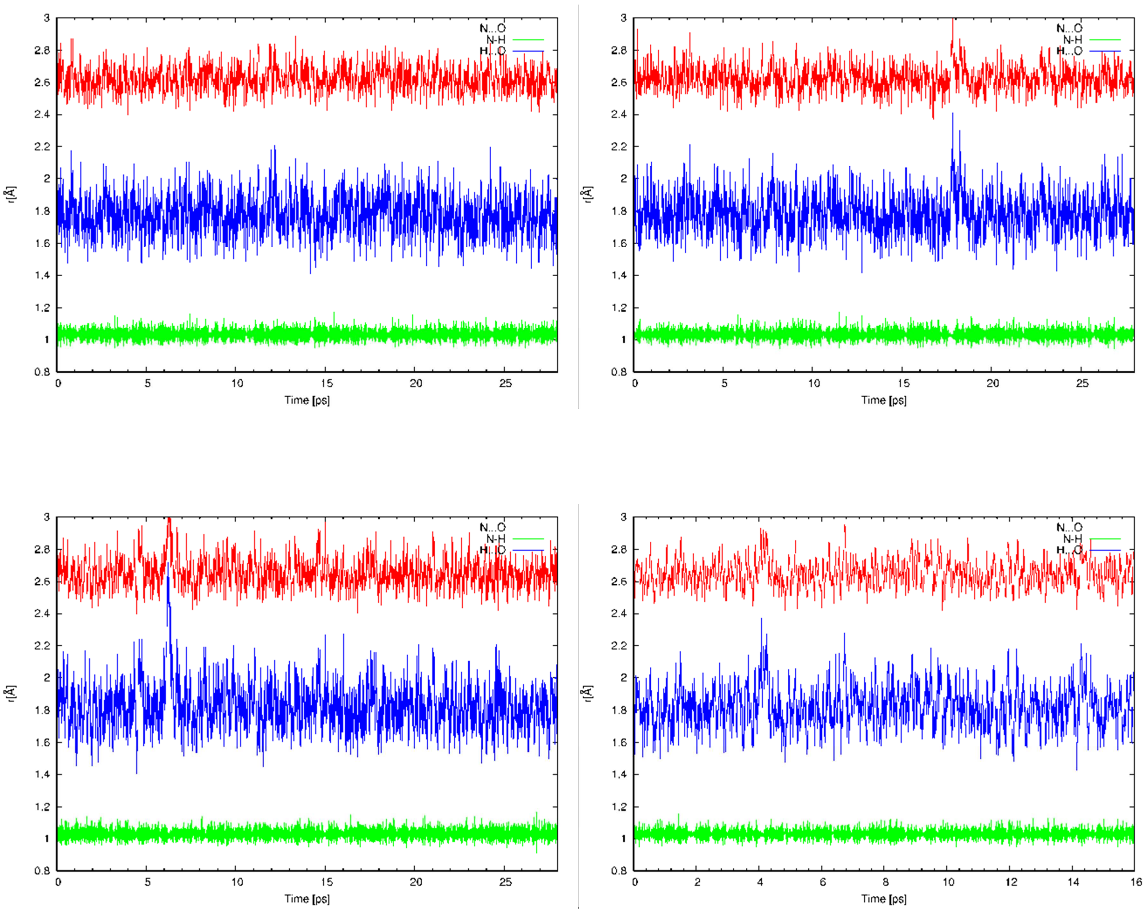


**Figure 1SI.** Time-evolution of the interatomic distances of the atoms involved in the hydrogen bridge formation. Results obtained from CPMD simulation within classical nuclear dynamics (left) and within inclusion of dispersion effects (right) in crystalline phase (upper graphs) and *in vacuo* (lower graphs).

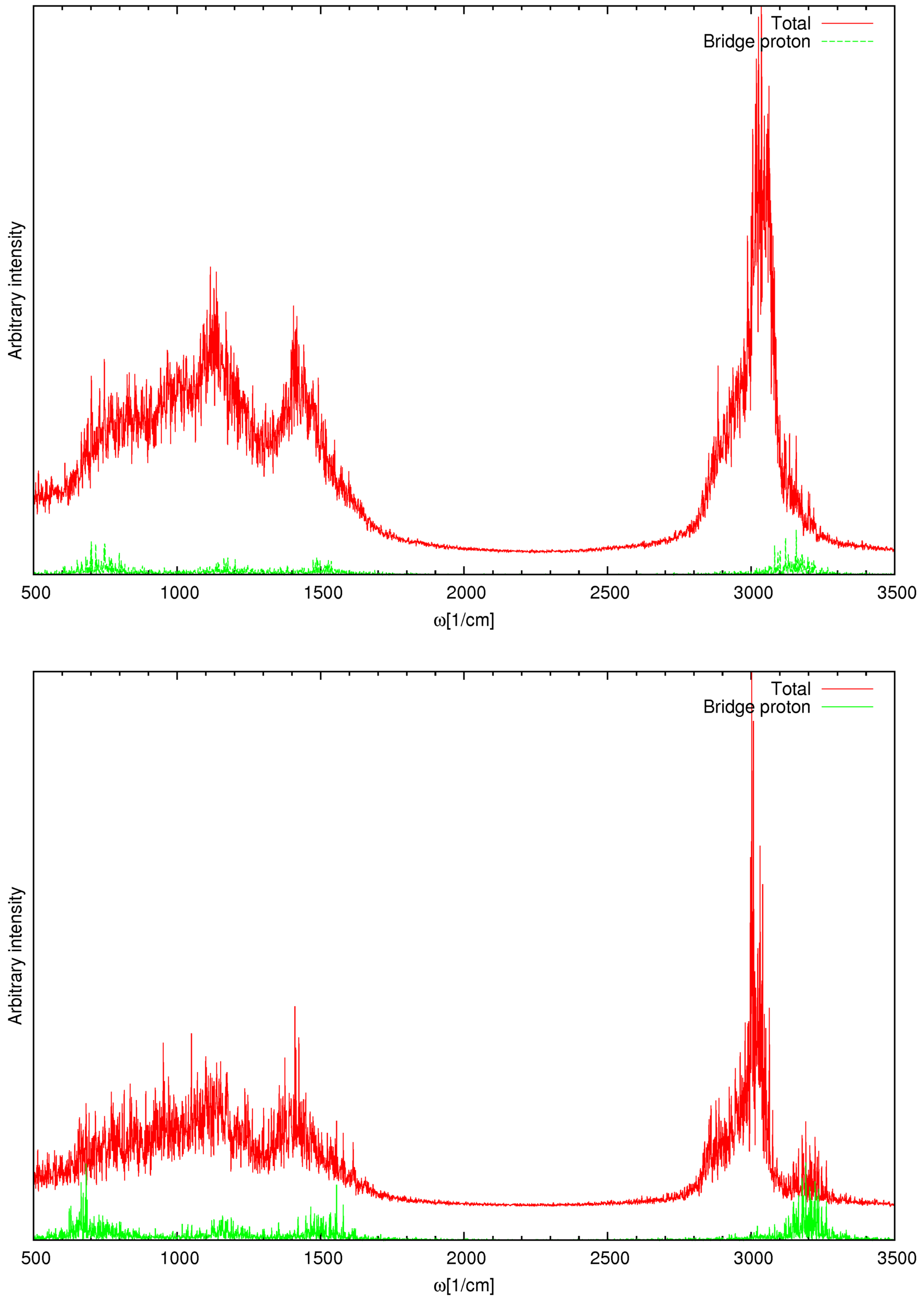


**Figure 2SI.** Power spectra of the atomic velocity of all atoms and the bridge proton of N-methyl-quinoline-2-carboxamide 1-oxide as a result of the CPMD simulations in the crystalline phase (upper spectra) and *in vacuo* (lower spectra). The intensities are given in arbitrary units, whereas the wavenumbers correspond to the actual vibrational properties of the studied compound.

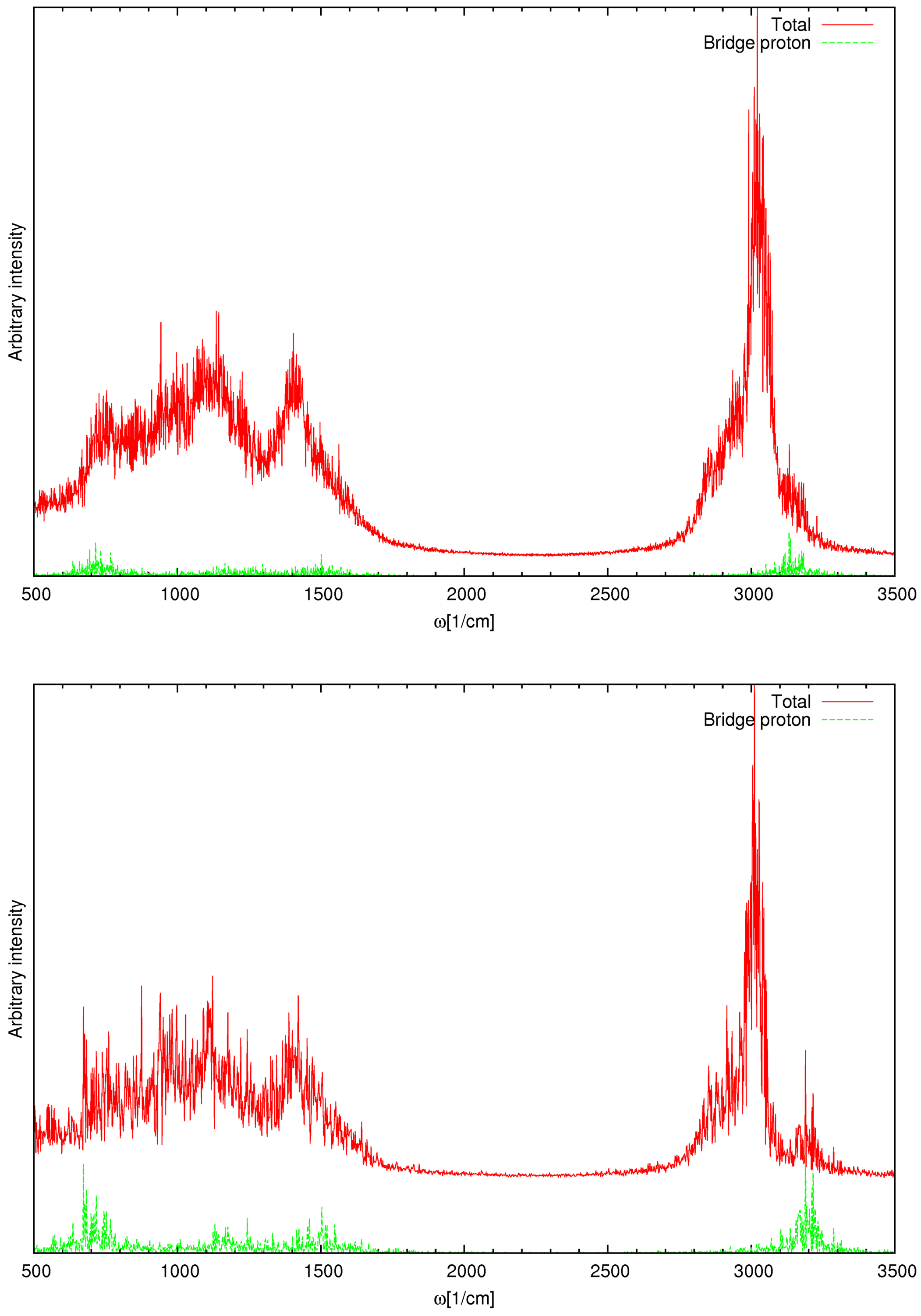


**Figure 3SI.** Power spectra of the atomic velocity of all atoms and the bridge proton of N-methyl-quinoline-2-carboxamide 1-oxide as a result of the CPMD simulations with Grimme's dispersion correction in the crystalline phase (upper spectra) and *in vacuo* (lower spectra). The intensities are given in arbitrary units, whereas the wavenumbers correspond to the actual vibrational properties of the studied compound.

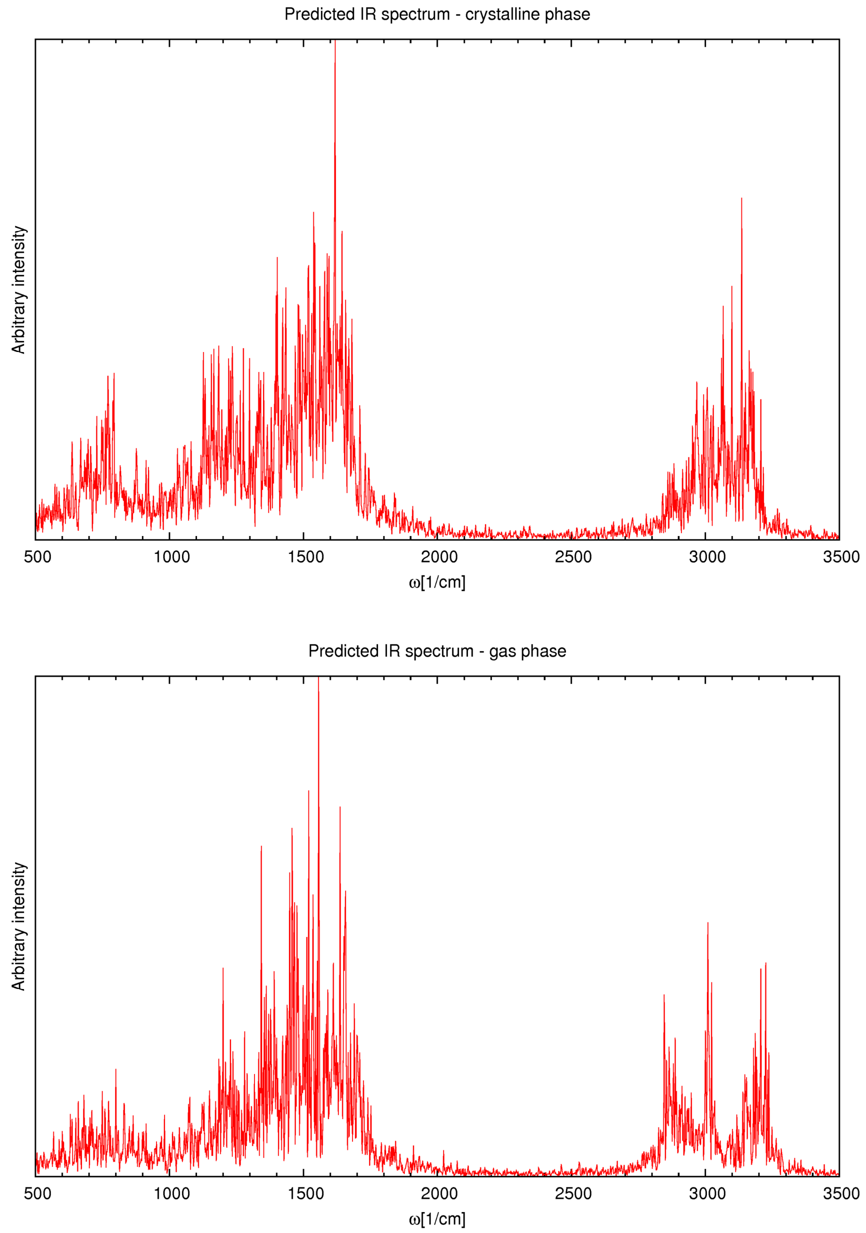


**Figure 4SI.** Predicted IR spectrum of N-methyl-quinoline-2-carboxamide 1-oxide. Results obtained from the CPMD simulation in the crystalline phase and *in vacuo*. Intensities, in arbitrary units, are proportional to the observable IR intensities.

**Table 1SI.** Comparison of the experimental X-ray (Ref. 12) and computed interatomic distances of atoms involved in the intramolecular hydrogen bond formation obtained for N-methyl-quinoline-2-carboxamide 1-oxide. Static DFT and CPMD in the crystalline phase and *in vacuo*.

| **X-ray** (Ref. 12) | **Metric parameters** | | | |
|---|---|---|---|---|
| | **N…O [Å]** | **N-H [Å]** | **H…O [Å]** | **N-H…O [°]** |
| | **2.598** | **0.907** | **1.842** | **139.23** |
| **Level of theory** | | | | |
| **B3LYP/6-311++G(d,p)** | 2.62803 | 1.01798 | 1.80290 | 135.538 |
| **B3LYP-D3/6-311++G(d,p)** | 2.62856 | 1.01753 | 1.80827 | 135.044 |
| **PBE/6-311++G(d,p)** | 2.62841 | 1.03090 | 1.76942 | 138.090 |
| **PBE-D3/6-311++G(d,p)** | 2.62719 | 1.02990 | 1.77991 | 136.817 |
| **wB97xD/6-311++G(d,p)** | 2.61612 | 1.01628 | 1.79587 | 135.111 |
| **CPMD (solid state) average** | 2.6219 | 1.0339 | 1.7752 | 136.84 |
| **CPMD (solid state with dispersion) average** | 2.6251 | 1.0339 | 1.7835 | 136.30 |
| **CPMD (gas phase) average** | 2.6521 | 1.0320 | 1.8232 | 135.34 |
| **CPMD (gas phase with dispersion) average** | 2.6498 | 1.0319 | 1.8196 | 135.40 |

**Ref. 12:** Kamiński R, Schilf W, Cmoch P, Dziembowska T, Woźniak K (2009) On structural and spectroscopic differences between quinoline-2-carboxamides and their N-oxides in the solution and solid state. J Phys Org Chem 22:857-871

**Table 2SI.** Comparison of the experimental (Ref. 12) and computed geometric parameters (crystalline phase and *in vacuo*) of the studied N-H…O hydrogen bond: investigations of the influence of the cutoff value on the geometric parameters.

| **X-ray** (Ref. 12) | **Geometric parameters** | | | |
|---|---|---|---|---|
| | **N…O [Å]** | **N-H [Å]** | **H…O [Å]** | **N-H…O [°]** |
| | **2.598** | **0.907** | **1.842** | **139.23** |
| **Cutoff value** | | | | |
| **Crystalline phase** | | | | |
| **70 Ry** | 2.61194 | 1.03448 | 1.74491 | 138.585 |
| **80 Ry** | 2.61317 | 1.03239 | 1.74896 | 138.484 |
| **90 Ry** | 2.61120 | 1.03096 | 1.74886 | 138.408 |
| **100 Ry** | 2.59881 | 1.03156 | 1.72474 | 139.728 |
| **110 Ry** | 2.60851 | 1.03012 | 1.74471 | 138.669 |
| **120 Ry** | 2.60781 | 1.02893 | 1.74671 | 138.473 |
| **Gas phase** | | | | |
| **70 Ry** | 2.61303 | 1.03493 | 1.74204 | 139.020 |
| **80 Ry** | 2.61872 | 1.03079 | 1.75330 | 138.835 |
| **90 Ry** | 2.61536 | 1.02981 | 1.74941 | 138.996 |
| **100 Ry** | 2.61758 | 1.02932 | 1.75435 | 138.729 |
| **110 Ry** | 2.61583 | 1.02994 | 1.74860 | 139.140 |
| **120 Ry** | 2.61889 | 1.02898 | 1.75678 | 138.639 |
| **Crystalline phase with dispersion correction** | | | | |
| **70 Ry** | 2.61599 | 1.03587 | 1.74729 | 138.648 |
| **80 Ry** | 2.61519 | 1.03254 | 1.75234 | 138.312 |
| **90 Ry** | 2.61262 | 1.03111 | 1.75058 | 138.362 |
| **100 Ry** | 2.61109 | 1.03041 | 1.74865 | 138.482 |
| **110 Ry** | 2.60938 | 1.03051 | 1.74445 | 138.768 |
| **120 Ry** | 2.61844 | 1.03004 | 1.75959 | 138.124 |
| **Gas phase with dispersion correction** | | | | |
| **70 Ry** | 2.62611 | 1.03359 | 1.76284 | 138.294 |
| **80 Ry** | 2.62234 | 1.03047 | 1.76148 | 138.335 |
| **90 Ry** | 2.61759 | 1.02940 | 1.75336 | 138.843 |
| **100 Ry** | 2.62086 | 1.02903 | 1.75977 | 138.517 |
| **110 Ry** | 2.62190 | 1.02863 | 1.76439 | 138.135 |
| **120 Ry** | 2.61899 | 1.02896 | 1.75721 | 138.601 |

**Ref. 12:** Kamiński R, Schilf W, Cmoch P, Dziembowska T, Woźniak K (2009) On structural and spectroscopic differences between quinoline-2-carboxamides and their N-oxides in the solution and solid state. J Phys Org Chem 22:857-871
